# Comparative Mitogenomics of Leeches (Annelida: Clitellata): Genome Conservation and *Placobdella*-Specific *trnD* Gene Duplication

**DOI:** 10.1371/journal.pone.0155441

**Published:** 2016-05-13

**Authors:** Alejandro Oceguera-Figueroa, Alejandro Manzano-Marín, Sebastian Kvist, Andrés Moya, Mark E. Siddall, Amparo Latorre

**Affiliations:** 1 Laboratorio de Helmintología, Departamento de Zoología, Instituto de Biología, Universidad Nacional Autónoma de México, Coyoacán, 04510, Mexico City, Mexico; 2 Research Collaborator, Department of Invertebrate Zoology, Smithsonian Institution. National Museum of Natural History, Washington D. C., United States of America; 3 Institut Cavanilles de Biodiversitat i Biologia Evolutiva, Universitat de València, Catedrático José Beltrán 2, 46008, Paterna, Valencia, Spain; 4 Department of Natural History, Royal Ontario Museum, 100 Queen’s Park, Toronto, ON, M5S 2C6, Canada; 5 Department of Ecology and Evolutionary Biology, University of Toronto, 25 Willcocks Street, Toronto, ON, M5S 3B2, Canada; 6 Área de Genómica y Salud de la Fundación para el Fomento de la Investigación Sanitaria y Biomédica de la Comunidad Valenciana (FISABIO), Avenida de Catalunya 21, 46020, Valencia, Spain; 7 Sackler Institute for Comparative Genomics, American Museum of Natural History, Central Park West at 79th Street, New York, NY, 10024, United States of America; University of Lausanne, SWITZERLAND

## Abstract

Mitochondrial DNA sequences, often in combination with nuclear markers and morphological data, are frequently used to unravel the phylogenetic relationships, population dynamics and biogeographic histories of a plethora of organisms. The information provided by examining complete mitochondrial genomes also enables investigation of other evolutionary events such as gene rearrangements, gene duplication and gene loss. Despite efforts to generate information to represent most of the currently recognized groups, some taxa are underrepresented in mitochondrial genomic databases. One such group is leeches (Annelida: Hirudinea: Clitellata). Herein, we expand our knowledge concerning leech mitochondrial makeup including gene arrangement, gene duplication and the evolution of mitochondrial genomes by adding newly sequenced mitochondrial genomes for three bloodfeeding species: *Haementeria officinalis*, *Placobdella lamothei* and *Placobdella parasitica*. With the inclusion of three new mitochondrial genomes of leeches, a better understanding of evolution for this organelle within the group is emerging. We found that gene order and genomic arrangement in the three new mitochondrial genomes is identical to previously sequenced members of Clitellata. Interestingly, within *Placobdella*, we recovered a genus-specific duplication of the *trnD* gene located between *cox2* and *atp8*. We performed phylogenetic analyses using 12 protein-coding genes and expanded our taxon sampling by including GenBank sequences for 39 taxa; the analyses confirm the monophyletic status of Clitellata, yet disagree in several respects with other phylogenetic hypotheses based on morphology and analyses of non-mitochondrial data.

## Introduction

Mitochondrial DNA sequences, alone or in combination with nuclear markers and morphological data, have been widely used to investigate the phylogenetic relationships of an extensive array of organisms (e.g. [1–5]), as well as in efforts to estimate biological diversity and identify specimens through the Barcode of Life initiative [6], which relies on a short fragment of the mitochondrial genome (*cox1*) in order to identify unknown specimens. With increased availability of complete mitochondrial genomes, in part due to the advancement of high-throughput sequencing, comparative studies targeting the differences in rates of evolution between genes, as well as gene rearrangements, gene duplication and gene loss are progressively feasible [7–9]. Whereas the timing and mode of these evolutionary events are quite well understood in mammals [10–12] and other model organisms [13,14], our knowledge concerning the mitochondrial genomic makeup of most invertebrate taxa is still limited. One such group is Annelida, a rather large phylum with more than 17,000 currently recognized species [15]. Once considered to be an easily diagnosable group given the serial homology of the segmented body plan (see [16]), modern phylogenetic hypotheses for Annelida have revealed that the phylum includes several groups without noticeable segmentation, such as Siboglonidae (= Pogonophora), Urechidae (Echiura) and Sipunculidae (Sipuncula) [17–23]. Whereas some other groups of organisms, such as arthropods and molluscs, display a relatively high number of rearrangements within mitochondrial genomes, gene order within Annelida has been hypothesized to be relatively well conserved [24–26]. Somewhat contrary to this hypothesis, recent investigations into the mitogenomic makeup of several annelids have revealed substantial disparity between taxa, especially when including early diverging lineages (see e.g. [27–30]). As a result of the bulk of taxonomic diversity within Annelida being contained within the paraphyletic class Polychaeta, most mitogenomic studies concern polychaete taxa. That is to say that there is notable paucity of comparative data for the remaining class Clitellata (including hirudineans, branchiobdellids, acanthobdellids and oligochaetes), which often forms a well-supported monophyletic group in contemporary phylogenetic analyses (but see [17]); in addition, representatives possess a clear morphological synapomorphy, the clitellum. This lack of data is most conspicuous when regarding the sequenced mitogenomes of Hirudinea (including more than 680 species, which exhibit a variety of morphological traits, feeding preferences and ecological functions [31,32]). In fact, only two complete (*Whitmania pigra* [Whitman 1886] and *Whitmania laevis* [Baird, 1869]) and one partial mitochondrial genome (*Helobdella robusta* [Shankland et al. 1992]) have so far been generated [33,34]. Note here that leech mitochondrial genomes available in public databases under the taxonomic labels *Hirudo nipponia* Whitman 1886 (GenBank accn. number: NC_023776 and KC667144), *Erpobdella octoculata* (Linnaeus 1758) (GenBank KC688270), *Whitmania acranulata* (Whitman 1886) (GenBank KC688271) and *Hirudinaria manillensis* Lesson 1842 (GenBank KC688268.1) were excluded from the present study. This is because a comparison between *cox1* sequences from these complete genomes and isolated *cox1* sequences from the same taxa (data not shown), confirm the notion that these mitogenomes are either derived from misidentified taxa or are a result of sequencing contaminations, as has previously been pointed out [35]. Most likely, the aforementioned mitogenomes belong to an unidentified *Whitmania* species. Additionally, after further investigation of these genomes, we concluded that they all share the same gene order as the *W*. *pigra* mitogenome, such that they would add no further data regarding (e.g.) genome rearrangements.

In the present study, we provide a detailed comparative investigation into the composition of mitochondrial genomes for three bloodfeeding glossiphoniid leeches: *Placobdella lamothei* Oceguera-Figueroa and Siddall 2008, *Placobdella parasitica* (Say 1824) and *Haementeria officinalis* De Filippi 1849. In addition, we place our results in a phylogenetic context using the 12 protein coding genes from 42 complete or nearly complete annelid mitochondrial genomes, using other representatives of Lophotrochozoa (Bryozoa and Brachiopoda) as outgroups.

## Materials and Methods

### Collection of specimens, sequencing, assembly and annotation of mitochondrial genomes

Specimens of *Haementeria officinalis* and *Placobdella lamothei* were collected in the Mexican states of Guanajuato and Estado de Mexico, respectively, under the collection permit SEMARNAT 12099/14 to AO-F. The two leech species are not listed as endangered or protected by Mexican government and are not CITES listed. Studying these species does not require approval of ethics committee. Voucher specimens were deposited in the Colección Nacional de Helmintos, at the Instituto de Biología, Universidad Nacional Autónoma de Mexico (CNHE Catalogue numbers 8621, 5679).

Reads from the mitochondrial genomes of *H*. *officinalis* (454 flx+) and *P*. *lamothei* (454 flx+ and HiSeq2000) were generated as a by-product of a sequencing effort focused on the non-cultivable bacterial endosymbionts of these organisms [36]. In short, 454 reads were filtered using PyroCleaner v1.3 [37] and the remaining reads were taxonomically assigned using PhymmBL v4.0 [38] with custom-added genomes of representatives from the class Insecta (*Atta cephalotes*, *Acyrthosiphon pisum*, *Drosophila melanogaster* and *Tribolium castaneum*), *Homo sapiens* GRCh37.p5, and the leech *Helobdella robusta*, in addition to their corresponding mitochondrial genomes. For *P*. *parasitica*, 454 flx+ titanium reads were recovered from the Short Read Archive (SRA) from NCBI (accession SRX101489) (see details in [39]). These data resulted from a previous study aimed at characterizing an endosymbiotic leech bacteria. The 454 reads were cleaned using PyroCleaner in order to remove duplicates, reads shorter than 100 bp, reads longer than 1000 bp and reads that had quality PHRED scores lower than 35. HiSeq2000 reads were cleaned from artifacts, right-tail clipped and scrutinized for length (minimum length of 50 bp) using the FASTX-toolkit v0.0.13.2 (http://hannonlab.cshl.edu/fastx_toolkit/). The standalone version of PRINSEQ v0.20.3 [40] was used to remove reads containing undefined nucleotides and perform de-duplication. For the reconstruction of *H*. *officinalis* (Genbank:JN850907) and *P*. *parasitica* (Genbank:AF003261) mitochondrial genomes, partial *cox1* sequences were retrieved from the nucleotide database from NCBI. For *P*. *lamothei*, a partial *cox1* sequence from a previous study [41] was used. A process of iterative assembly (mapping and extension of contigs) starting with the partial *cox1* nucleotide sequences using MIRA v3.4.1 [42] was performed for each of the genomes, missing only part of the long non-coding regions. For *P*. *lamothei*, HiSeq2000 reads were used to perform gap-filling with GapFiller v1.9 [43] and correction of nucleotides using Polisher v2.0.8 (available for academic use from the JGI http://www.jgi.doe.gov/software/), although no errors were detected. Annotations were performed using MITOS [44] and were originally used to identify tRNAs, rRNA regions and putative coding gene regions. From there, we identified putative start codons on the basis of them immediately following the 3' ends of other coding sequences, tRNAs or rRNAs, with subsequent comparison against other Clitellata genomes. Stop codons were determined as follows: 1) canonical stop codons, which did not overlap with tRNAs or 2) partial stop codons, where tRNA-adjacent T or TA bases have been transcriptionally modified to add alanine residues to the 3' of the mRNA to complete the stop codon. If none of the above conditions were met, the gene was annotated as overlapping the adjacent feature in its 3' end. All annotated sequences are deposited in the International Nucleotide Sequence Database Collaboration [45] (INSDC; http://www.insdc.org) under the previously mentioned accession numbers.

### Mitochondrial gene order

A set of 34 previously sequenced mitochondrial genomes of annelids, with almost complete protein-coding gene sequences, was retrieved from the NCBI nucleotide database (keywords: “annelida” and “mitochondrion”). Note that the genome of *Whitmania laevis*, which was recently published [35] has been shown to match the gene order of *Whitmania pigra* (see above) and, therefore, this taxon was not included in the present study. Visual display of genes was performed in R [46] using the package genoPlotR v0.8.2 [47] and edited in Inkscape v0.91 (https://inkscape.org/en/).

### Phylogenetic analysis

The previously described set of annelid mitochondrial genomes, plus an additional 6 complete mitochondrial genomes from representatives of Bryozoa and Brachipoda (outgroups) were used for the phylogenetic analyses. Accession numbers and taxonomic classification for all mitochondrial genomes retrieved are available in S1 Table. All the retrieved sequences were then re-annotated using MITOS with subsequent manual curation as explained above. From the NCBI-formatted files, amino acid sequences in FASTA format where extracted and aligned using MAFFT v7.220 [48] with the '—localpair' option. Then, Gblocks v0.91b [49] was used with the options '-t = p -b2 = 21 -b3 = 10 -b4 = 5 -b5 = h' to permit smaller blocks. The rational behind the use of amino acids instead of nucleotides resides in the difficulty of accurately modeling saturations, which is particularly exacerbating when working with distantly related taxa and/or fragmentary taxon sampling. The use of Gblocks reduced the final alignment (an average of 31% for each gene), resulting in a matrix with a smaller number of gaps. The resulting amino acid sequence alignments where then fed into ProtTest v3.4 [50] to search for the best-fit model of evolution for each gene. ProtTest suggested the following models: MtArt+I+G+F for *atp6*,*cox2*, *nad1*, *nad2*, *nad4*, *nad5*; MtArt+G for *cytb*, *cox3*, *nad3*, *nad6*; LG+I+G+F for *cox1*; MtREV+I+G+F for *nad4L*. The MtArt was implemented in MrBayes v3.2.4 [51] through the use of the option prset aarevmatpr for the replacement matrix and prset statefreqpr for the amino acid frequencies. For the Bayesian analyses, two independent runs, each with four chains (three "heated", one "cold") were run for 3,000,000 generations discarding the first 25% as burn-in, even though convergence was reached before 30,000 generations. The tree was then loaded into FigTree v1.4.1 (http://tree.bio.ed.ac.uk/software/figtree/) and edited with Inkscape v0.91 (https://inkscape.org/en/). For the parsimony analyses, a new technology search was performed in TNT [52] treating gaps as a fifth state and using 1000 initial addition sequences, five rounds of ratcheting [53] and three rounds of tree fusing [54] after the initial Wagner tree builds, and requiring that the minimum length tree be found a total of ten times. The resulting trees were then returned to TNT and used as starting trees for a new TBR branch swapping search using the command ‘bbreak’. Bootstrap support values were calculated from 1000 pseudoreplicates with the same settings as mentioned above.

## Results & Discussion

### Mitochondrial genomes of *H*. *officinalis*, *P*. *lamothei* and *P*. *parasitica*

#### Genomic organization

Mitochondrial genomes of *H*. *officinalis*, *P*. *lamothei* and *P*. *parasitica* were assembled into single contigs of 14,849bp (linear) (ENA accn. number: LT159848), 14,909bp (linear) (ENA accn. number: LT159849) and 15,190bp (circular; closed) (ENA accn. number: LT159850), respectively. Although the first two genomes were not closed, they contain all 37 mitochondrial genes and are only lacking a very short part of the putative control region (pCR), when compared to the remaining taxa (although its length when complete is still unknown). All 37 genes are encoded on the same strand in all three mitochondrial genomes, which all contain a large non-coding region between the genes *trnR* and *trnH*–this region corresponds to the pCR. The position of the pCR is variable across the diversity of annelid genomes but fully conserved among representatives of Clitellata, with *Whitmania pigra* showing the shortest length (80bp) and highest A+T content (78.75%) (S2 Table).

#### Protein-coding and RNA genes

**Fig 1:**
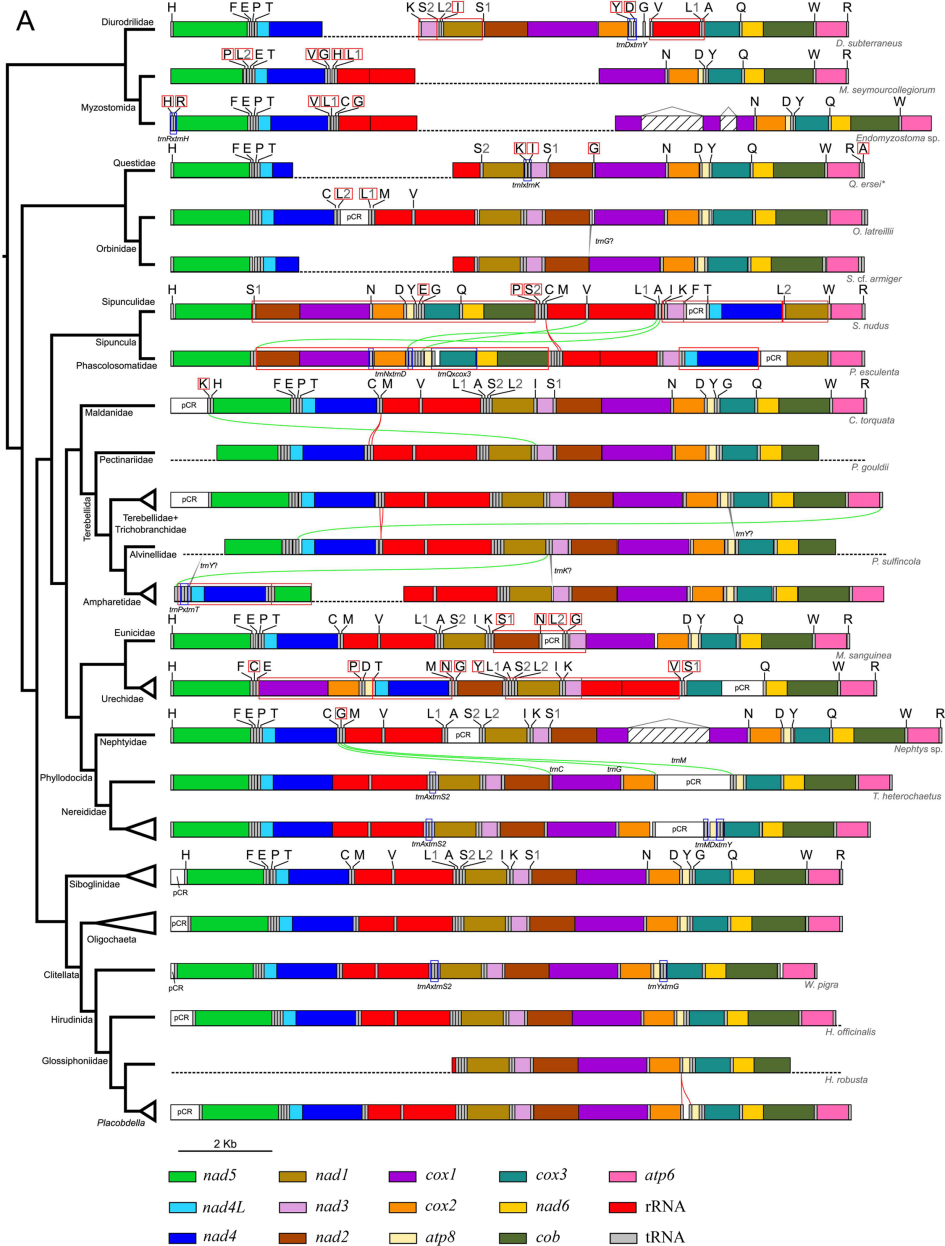
Gene order from available mitochondrial genomes of Annelida. The phylogeny on the left was inferred by Bayesian methodology. Colors indicate each of the 13 different protein-coding genes present in the mitochondrial genomes. Genes are scaled to real length. Green lines connecting one horizontal bar to another track the position of tRNA gene rearrangements. Red lines are used to indicate duplication of tRNA genes. Red boxes around tRNA gene-names indicate putative rearrangements of tRNA genes relative to the mutual gene order for Clitellata. Red boxes around blocks of genes highlight syntenic regions, which may have been rearranged as a single unit. Blue boxes around blocks of genes denote inversions and, finally, incomplete or missing data are denoted by dashed lines. Names of RNA genes are denoted only when there has been a change in sequence, if RNA genes are not denoted, their sequence is equal to the closest sequence above for which genes are denoted. In some cases, however, minor rearrangements are present and these are then denoted with the names of the RNA genes involved and separated by “x”. For example, *trnA*x*trnS2* indicates that the *trnA* and *trnS2* genes have switched places. Gapped insertions inside *cox1* in *Endomyzostoma* sp. and *Nephtys* sp. indicate group II introns.

Protein-coding genes for the three leech genomes sequenced in this study were initially identified using MITOS, an integrated platform for mitochondrial genome annotation. The order of all 13 protein-coding genes is identical to that of previously sequenced mitochondrial genomes of clitellates (Fig 1). Several instances of tRNA gene rearrangements and duplications of tRNA genes were detected across the tree. Importantly, a *trnD* duplication was detected in the two *Placobdella* species included in the analysis (see below). Subsequently, we investigated the nucleotide compositions of all start and stop codons for the protein-coding genes (S3 Table). Most of these genes present putative incomplete stop codons (T or less frequently TA) but these are likely completed by post-transcriptional addition of 3'-end alanine residues [55–57]. These putative incomplete stop codons are flanked at the 3'-end by the 5'-end of tRNA genes or, less commonly, by another protein-coding gene. Much like other members of Clitellata, the 3'-end of the *nad4L* gene overlaps the 5'-end of the *nad4* gene. This feature is conserved in all Annelida mitochondrial genomes sequenced so far, except for that of *Clymenella torquata* (Leidy 1855)–this is probably a product of a misannotation in RefSeq. Regarding the start codons for the newly sequenced mitogenomes, all protein-coding genes share the canonical ATG start codon, except for that of *cox3*, where we observed a Glossiphoniidae-specific shift to the alternative start codon TTG. In *P*. *lamothei*, we also observed a shift from ATG to GTG in the gene *nad1*. This event seems to be something that has arisen independently in this particular species since its relative *P*. *parasitica* presents the canonical ATG.

In all three newly sequenced mitogenomes, as well as those of previously sequenced clitellates, rRNA genes are located between the *trnM* and the *trnL1* genes, and are only separated by the mid-rRNA placement of the *trnV* gene. The tRNA genes are relatively conserved in sequence order and structure in all three genomes, except for the remarkable case of the *trnR* gene in *P*. *parasitica* (S1 Fig). In this organism there is a 20 bp non-coding region between the *atp6* and *trnR* genes. This tRNA still preserves a typical cloverleaf structure, pointing towards the validity of the annotation of this apparent *P*. *parasitica*-specific change. Finally, putative overlap between tRNA genes with adjacent tRNA or protein-coding genes seems to be perfectly conserved in the three genomes sequenced here (Fig 1).

#### *Placobdella*-specific trnD duplication

**Fig 2:**
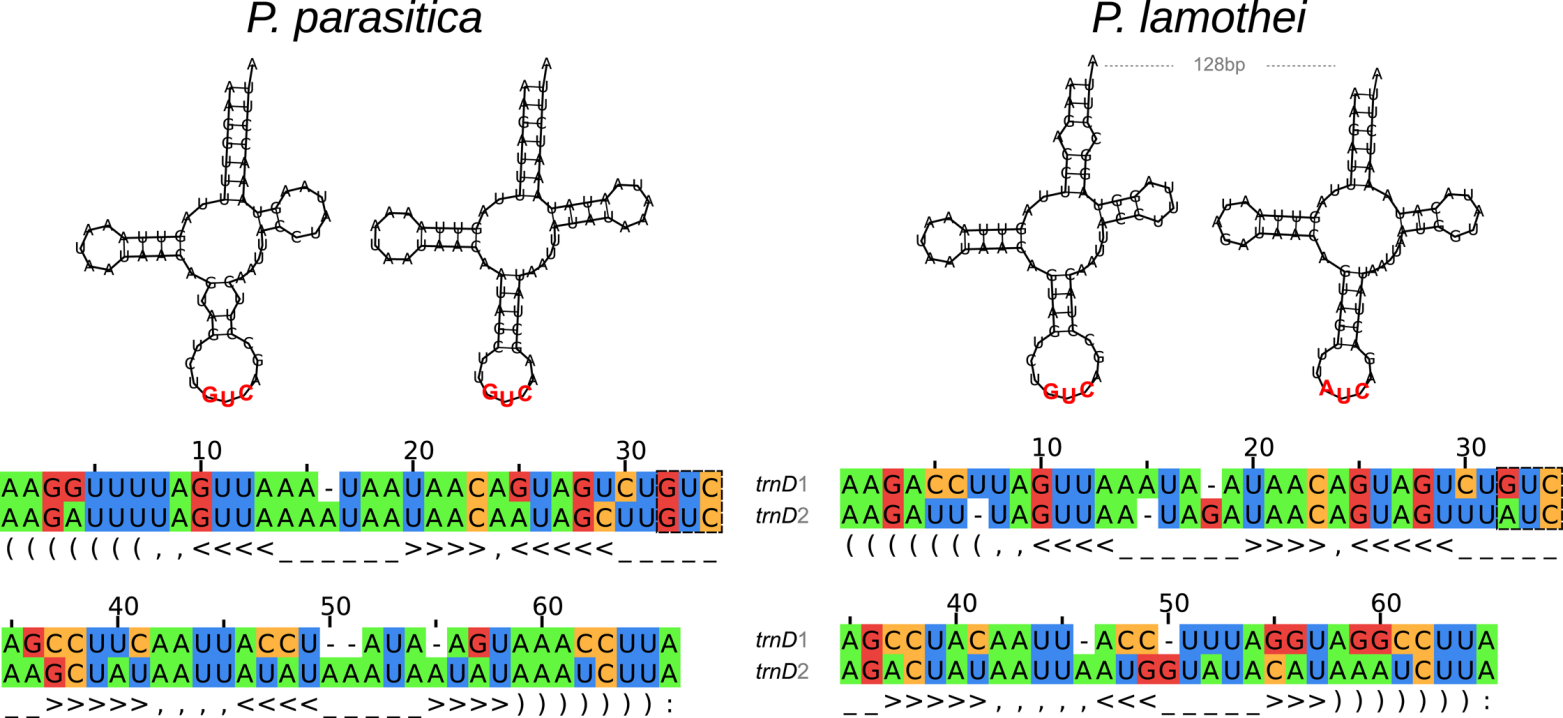
*trnD* duplication in *Placobdella*. Top: Secondary structures of *Placobdella trnD* genes as calculated by MITOS. Bottom: *trnD* sequences aligned against the 'Metazoa_D' model from MiTFi v0.1 [77] using cmalign from Infernal v1.1rc4 [78]; sequence alignments took into account the secondary structure of the *trnD* genes. Note that gaps in the alignment occur in the loop structures or the *trnD*’s.

Surprisingly, our results indicate a *Placobdella*-specific duplication of the *trnD* gene (Figs 1 and 2). We were unable to find this duplication in any other completely sequenced annelid mitochondrial genome; we propose that this duplication is restricted to the *Placobdella* clade. Even more surprising is the fact that these two *trnD* genes seem to be potentially functional, inasmuch as they both preserve a cloverleaf structure and strong sequence similarity when compared to the remaining copy in the same species (Fig 2). Importantly, whereas there is no overlap or intergenic region between these two tRNA genes in *P*. *parasitica*, we found a 128 bp non-coding region (the longest such region except for the pCR) in *P*. *lamothei*. Using the mfold [58] webserver, we were able to find two small stem-loop structures in this region, the functions of which (if any) are unknown. It is also noteworthy that, in the case of *P*. *lamothei*, we found a change in the anticodon of the second *trnD* gene from GUC to AUC (codons GAC and GAT, respectively). Given this change, we explored the amino acid composition of the proteins from Clitellata mitochondrial genomes, finding no statistical difference in the amino acid frequency for any proteome (Kruskal-Wallis paired test: chi-squared = 7.2333, df = 7, p-value = 0.405). We also analyzed, using the same set of proteins, the codon usage for the two alternative codons for aspartic acid (GAC and GAT) (Table 1). Using Fisher's exact test, we determined that there was indeed a difference in codon usage among the different mitochondrial genomes for Clitellata (p-value = 1.455E^-15^). By removing one or more terminals from all possible combinations, progressively, until reaching subgroups with no significant statistical differences among their members, we found two groups with similar codon usage: (i) Oligochaeta (Fisher's exact test: p-value = 0.1508), (ii) *W*. *pigra*, *H*. *officinalis* and *P*. *parasitica* (Fisher's exact test: p-value = 0.3999). The inclusion of *P*. *lamothei* into either one of these groups gave statistically significant differences (with i, Fisher's exact test: p-value = 0.01662; with ii, Fisher's exact test: p-value = 0.006462). This difference in codon usage in *P*. *lamothei* could be evidence for the actual functionality of the two *trnD* genes, as the shift in codon usage may allow a more balanced utilization in the anticodons.

**Table 1:** Codon usage for aspartic acid in Clitellata mitochondrial genomes. Number of codons present in proteins used to code aspartic acid in Clitellata mitochondrial genomes. Even though all Hirudinea, except for *P*. *lamothei*, have a strong bias for the use of the GAT codon, they exclusively code for the *trnD* gene with a GUC anticodon.

Organism	GAC	GAT	GAT	Ratio (GAC/GAT)
*Lumbricus terrestris*	34	40	64.42	0.85
*Perionyx excavatus*	42	32	75.00	1.31
*Tonoscolex birmanicus*	41	28	76.64	1.46
*Amynthas aspergillus*	43	28	71.68	1.54
*Metaphire vulgaris*	32	40	74.38	0.80
*Whitmania pigra*	12	78	78.75	0.15
*Haementeria officinalis*	8	58	~69.18	0.14
*Placobdella parasitica*	14	56	~70.10	0.25
*Placobdella lamothei*	27	45	75.63	0.60

Nevertheless, while all genomes of Hirudinea have a bias towards using the GAT codon, they still use the GUC anticodon to code for *trnD* genes (with the exception of *P*. *lamothei*). This evinces the fact that the specific tRNA anticodon is not directly influencing the codon usage for aspartic acid in *W*. *pigra*, *H*. *officinalis* and *P*. *parasitica*. It is worth noting that, within Annelida, there are at least two other cases of apparent functional tRNA gene duplication—one within the sipunculids (*Phascolosoma esculenta* [Chen and Yeh 1958]) [59] and one within Terebellida (*Pectinaria gouldii* [Verrill 1873], *Pista cristata* [Müller 1776] and *Terebellides stroemi* [Sars 1835]) [26]. No functional explanation for these duplications has yet been provided.

It is worth noting that both *trnD* copies are located on the *cox2*—*atp8* junction, the very same position proposed as a hotspot for mitochondrial rearrangements in hymenopterans [60]; whether or not the occurrence of duplications at this junction has a biological meaning remains to be investigated in detail. This finding, apparently restricted to *Placobdella* species, represents a new case for the study of the mechanisms of “duplication/loss”, in which a fragment of the mitochondrial genome is duplicated by slipped-mispairing during replication, followed by the slow conversion of supernumerary genes to pseudogenes through random replication errors. If the duplication of the *trnD* occurred in the last common ancestor of the genus, estimated to exist around 250 mya. [61], each one of the more than 20 species of the genus represents an independent case for the study of disposal of supernumerary genes, as seems to be an explanation for mitogenomic stability. Otherwise, if independent duplications of *trnD* are inferred based on phylogenetic analyses of the genus, the hypothesis of the *cox2*—*atp8* junction as a hotspot should be explored in detail (see [62]).

### Mitochondrial gene-order conservation across Annelida

**Fig 3:**
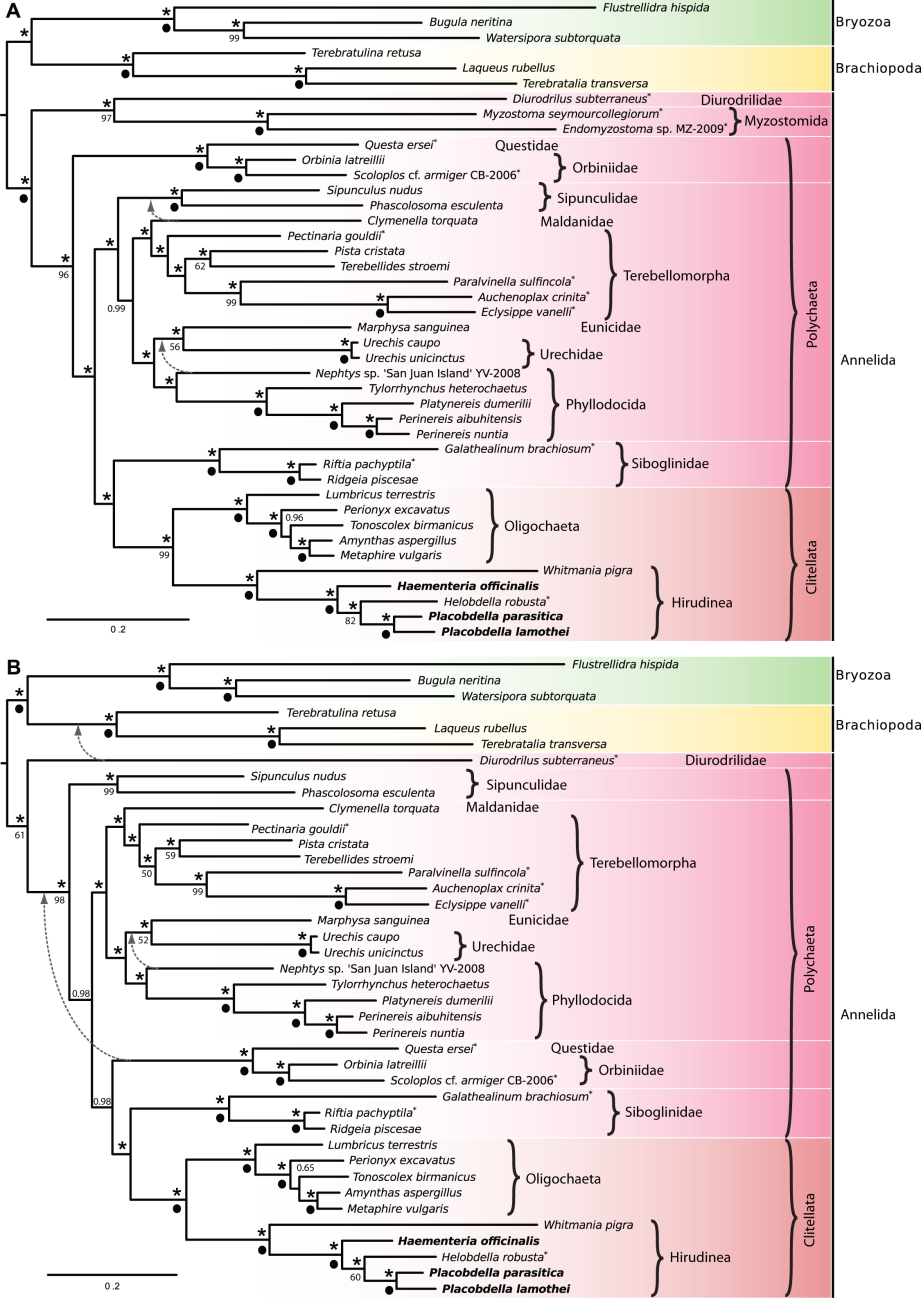
Phylogenetic relationships of Annelida. Bayesian phylogenetic reconstruction as inferred by MrBayes with Myzostomida included, A, and excluded, B. Values above nodes are Bayesian posterior probabilities, and those below the nodes are parsimony bootstrap support values above 50%; asterisks denote maximum posterior probabilities and black circles denote maximum parsimony bootstrap support. Taxa in bold were sequenced for the present study and broken lines indicate the alternative positioning of the respective taxa in the single most parsimonious trees.

Mitochondrial gene order within Annelida remains contentious; both a high level of stasis [25,59,63] and relatively high amount of rearrangements (e.g. [28,29,64]) has been reported for select taxa. In a comprehensive overview of the phylum, Weigert et al. [30] suggest that major mitogenomic rearrangements are confined to early diverging lineages, whereas representatives of Pleistoannelida, including Errantia and Sedentaria (*sensu* [65]) show high conservation of gene order. To shed further light on this issue, we utilized our phylogenetic inferences as guides and analyzed the rearrangements that have occurred in the different taxa (Fig 1). Gene order (only the 13 protein codifying genes) in Clitellata is identical to that present in Siboglinidae, Nereididae, Nephtyidae, Alvinellidae, Terebellidae + Trichobranchidae, Pectinariidae, Maldanidae, Orbinidae, Questidae and Myzostomida, and is also identical to the putative ground pattern of Pleistoannelida as described by Weigert et al. [30]. Changes in mitochondrial architecture are restricted to Eunicidae, Urechidae, Ampharetidae, Sipunculidae and Diurodrillidae and are discussed in comparison with the putative ground pattern for Pleistoannelida. In our analyses, Diurodrilidae by itself (Fig 3B) or as a clade together with Myzostomida (Figs 1 and 3A) places as the sister group to the remaining annelids and shows two modifications in its mitochondrial genome gene order, the first is a change in the order of *nad1* and *nad3* and the second is a new allocation of the rRNA. By contrast, the two representatives of Myzostomida both display the same protein coding gene order as the ground pattern for Pleistoannelida (Fig 1). Whereas Myzostomida seems to only show rearrangements of tRNA genes, Sipunculidae (also an early diverging lineage; Fig 3B) displays large rearrangements involving the translocation of various multigene syntenic blocks (*nad2*–*cob* switch location with *nad4L* and *nad4*). In addition, *nad1* also is reallocated. In corroborating the findings of Weigert et al. [30], we also observed identical gene order stasis within some distinct and well-defined groups. Within Terebellomorpha and Maldanidae, gene order is perfectly conserved, with the exception of a relocation of the *trnK* gene in *C*. *torquata* and a secondary loss of the *trnM* gene duplication in Ampharetidae; the latter taxon also portrays a seemingly more derived gene order, having rearranged the syntenic block *nad4L*–*nad4* for *nad5*. Within Phyllodocida, *Nephtys* sp. displays the peculiarity of having a group II intron inserted in the *cox1* gene [66]. Interestingly, this is also the case for the included species of *Endomyzostoma*. Nereididae shows a translocation of the *trnC*, *trnG* and *trnM* genes as well as an inversion of the *trnA* and *trnS2* genes when compared to *Nephtys* sp. Within the Questidae/Orbiniidae clade, *Questa ersei* Jamieson and Webb, 1984 shows an apparent lineage-specific inversion of the *trnI* and *trnK* genes–whether or not other species of Questidae share this is not known due to the lack of comparative data. In addition, *Scoloplos* cf. *armiger* shows a translocation of the *trnG* gene to an undetermined region between the *nad4* and *rrnL* gene. Lastly, the Siboglinidae/Clitellata clade recovered here, notwithstanding the phylogenetic method used or inclusion of Myzostomida, displays perfect synteny in mitochondrial gene-order, the same order as that observed for Questidae, Oribiniidae, Phyllodocida, Maldanidae, Terebellomorpha and matching the gene order recovered for most annelids [67]. The modified gene order within Urechidae (*cox1–atp8*; *nad1–nad2* and rRNA reallocations) and Eunicidae (*nad2* and *nad3* show switched positions) contradicts, to some extent, the finding by Weigert et al. [30] that major mitogenomic rearrangements are confined to early diverging lineages.

### Phylogenetic analyses

Beyond placing the newly sequenced taxa in a molecular phylogenetic framework, based on the mitochondrial genomes, the present study also capitalized on the assembly of the main dataset for the addressing of phylogenetic relationships across Annelida. A dataset consisting of 30 complete and 12 partially sequenced annelid mitochondrial genomes was assembled, including representatives of Myzostomida, Urechidae and Sipunculidae [18,19,21,22,27,63,68]. From these genomes, we generated a concatenated alignment of amino acids that was then used both for a partitioned Bayesian phylogenetic reconstruction and parsimony analysis. Because the inclusion of Myzostomida has been shown to possibly create discrepancies due to long-branch attraction [67], we decided to run two independent phylogenetic analyses, including and excluding myzostomids–the datasets are here referred to as the 42-taxa dataset (including Myzostomida) and the 40-taxa dataset (excluding Myzostomida). Both parsimony analyses resulted in single most parsimonious trees; 21,874 steps were recorded for the 42-taxa dataset with a retention index (RI) of 0.47 and consistency index (CI) of 0.46, and for the 40-taxa dataset, the single most parsimonious tree was 21,594 steps with an RI of 0.48 and a CI of 0.47. Two minor discrepancies exist between the Bayesian and the parsimony trees using the 42-taxa dataset. In the Bayesian tree, the sipunculids place as sister to a clade that includes most of the polychaetes (Bayseian posterior probability [PP] = 1.00), whereas in the parsimony tree, sipunculids is sister to *Clymenella torquata* (bootstrap support value [BS] = <50%). Moreover, *Nephtys* sp. places as the sister group of the remaining phyllodocids (PP = 1.00) in the Bayesian tree (Urechidae + Eunicidae places as the sister to this group), whereas the taxon places as sister to Urechidae+Eunicidae (BS = <50%) in the parsimony tree (Fig 3A). For the 40-taxa dataset, some major discrepencies are recovered when comparing the trees from the different analyses (Fig 3B). Most importantly, *Diurodrilus subterraneus* Remane 1934 is recovered as the sister group to Brachiopoda in the parsimony tree (BS = 61%), whereas its placement as the sister group to the remaining annelids is maintained in the Bayesian reconstruction (PP = 1.00). Oddly enough, the placement of Questidae+Orbiniidae changes rather dramatically in the Bayesian analysis when excluding the myzostomids as it is recovered as the sister to Siboglinidae+Clitellata (PP = 1.00). To shed light on this issue, future studies should focus on increasing the sampling of annelid taxa, as it has been shown that the inclusion of potential sister-taxa can minimize the effects of long-branch attraction in phylogenetic reconstructions [69,70].

Regarding the three glossiphoniid leeches sequenced for the present study, these form a monophyletic group with the partially sequenced *Helobdella robusta* (PP = 1.00 and BS = 100% for both datasets) and this group, in turn, places as the sister taxon to Arhynchobdellida (PP = 1.00 and BS = 100% for both datasets), which is here represented by the *W*. *pigra*. Interestingly, *H*. *officinalis* is recovered as the sister group to an *H*. *robusta*+*Placobdella* clade (PP = 1.00 for both datasets; BS = 82% and 84% for the 42-taxa dataset and 40-taxa dataset, respectively), which contradicts previous findings in which *Haementeria*+*Helobdella* are part of a sister radiation of *Placobdella* [71–73]. However, our results are most likely affected by shallow taxon sampling; the inclusion of more fully sequenced mitochondrial genomes for leeches may revert this result. It is also important to note that the included mitochondrial genome of *H*. *robusta* is incomplete and a substantial amount of missing data was present for this taxon, which may or may not alter the results. The phylogenetic positioning of *H*. *robusta* (a non-blood feeder) would further support the idea that *H*. *robusta* has secondarily lost the capacity for feeding on blood, along with the loss of its bacteriome and related endosymbiotic bacteria, traits that are present in species of both *Haementeria* and *Placobdella* [61]. Despite the paucity of available mitochondrial genome data for leeches, the current study represents the first phylogenetic analysis of full genomes that includes representatives of both leech orders (Rhynchobdellida and Arhynchobdellida). Considering that the gene order among the leeches included here is fully conserved, we hypothesize that no major rearrangements in the gene order and composition will be found after inclusion of more species.

The phylogenetic hypotheses presented here disagree to a large extent with some contemporary hypotheses. For example, contrary to Golombek et al. [67] and Weigert et al. [30], *Diurodrilus subterraneus* was here recovered as an early diverging lineage in all trees but in some of them with low support (PP = 1.00 for both datasets; BS = <50% and 61% for the 42-taxa dataset and the 40-taxa dataset, respectively) (Fig 3A and 3B). Furthermore, by virtue of the urechids nesting within Errantia, and Errantia nesting within Sedentaria (both placements with maximum posterior probabilities, but with much lower parsimony bootstrap support, 52% and <50%, respectively), Errantia and Sedentaria were never recovered as monophyletic. Beyond major inconsistencies regarding Errantia and Sedentaria, there are numerous minor differences in the branching patterns (cf. Fig 3 and Weigert et al. [30]: Fig 3) and seeing as the better part of these datasets overlap (with the exception of our use of Gblocks), these differences seem to be attributable to some specifics of the analyses and/or slightly different terminals included. For example, only *Helobdella robusta* and *Hirudo nipponia* were included in the datasets analyzed by Weigert et al. [30], and only one of these was included in each of the analyses performed by Weigert et al. [30]. Although the taxon sampling for Hirudinea was improved for the present analyses, we lack the inclusion of more “basal branching” members, which might also impact the phylogenetic inference. Regardless, the varying topologies in contemporary leech mitogenome analyses may simply indicate sensitivity to the elevated substitution rates for both amino acids and nucleotides, inherent in mitochondrial genomes [74]. Beyond this, recent investigations into the mitochondrial genomes of bilaterians suggest that mitochondrial data alone may not be fit for phylogenetic reconstructions inasmuch as the inversion of genes, changes in replication orders and compositional differences (e.g. AT-biases) all affect the resulting phylogeny [75,76]. In light of the discrepancies between our trees and previously published hypotheses, our results seem to substantiate this claim.

## Conclusions

As shown in Fig 1, genome architecture is conserved among the currently sequenced mitochondrial genomes of clitellates. Among other things, conservation of gene order is perfect, with the exception of two inversions found in *W*. *pigra* involving the tRNA gene-pairs *trnA*-*trnS2* and *trnY*-*trnG*. We also discovered a *trnD* gene duplication, which seems to be restricted to species of *Placobdella* but, nevertheless, we were unable to identify an explanation as to why this duplication occurred and has been maintained–it could be argued that not enough time has elapsed since the duplication event for it to be selectively purged. Additionally, by analyzing the different genome rearrangements of the currently available annelid mitochondrial genomes, one can find distinct inversions or translocations, which are specific to the different taxa.

## Supporting Information

S1 Fig tRNA structures of Clitellata mitochondrial genomes. tRNA structures of Clitellata tRNA genes as predicted by MITOS and manually curated. At the top of each set of blocks, dendograms showing the phylogenetic relationship of the organisms as calculated in the bayesian phylogenetic reconstruction. At the left of each individual block, the tRNA gene name indicated in bold italic letters with the anticodon sequence between parenthesis. On the tRNA structures, red letters indicate the anticodon, grey parallelograms indicate base pairs that overlap another gene sequence. In a blue block, the *Placobdella*-specific *trnD2* gene is shown with its two possible anticodons. Missing data for *H*. *robusta* are due to the lack of comparative data.
(PDF)

S1 File Concatenated alignment including myzostomids. Concatenated amino acid alignment used for phylogenetic reconstruction including myzostomids. The alignment was “cleaned” using Gblocks and is in nexus format.
(NEX)

S2 File Concatenated alignment excluding myzostomids. Concatenated amino acid alignment used for phylogenetic reconstruction excluding myzostomids. The alignment was “cleaned” using Gblocks and is in nexus format.
(NEX)

S1 Table Accession numbers and taxonomic classification for all mitochondrial genomes used in this study. (XLSX)

S2 Table General characteristics of putative control regions (pCRs) of mitochondrial genomes of Annelida. (XLSX)

S3 Table Start and stop codons for all 13 protein-coding genes from the mitochondrial genomes of Clitellata. (XLSX)
