# Supplementary material for: Comparative Mitogenomics of Leeches (Annelida: Clitellata): Genome Conservation and Placobdella-Specific trnD Gene Duplication
Source: PLoS One. 2016 May 13;11(5):e0155441. doi: 10.1371/journal.pone.0155441 (PMC4866719; doi:10.1371/journal.pone.0155441)
Supplement: S1 Fig — tRNA structures of Clitellata tRNA genes as predicted by MITOS and manually curated. At the top of each set of blocks, dendograms showing the phylogenetic relationship of the organisms as calculated in the bayesian phylogenetic reconstruction. At the left of each individual block, the tRNA gene name indicated in bold italic letters with the anticodon sequence between parenthesis. On the tRNA structures, red letters indicate the anticodon, grey parallelograms indicate base pairs that overlap another gene sequence. In a blue block, the Placobdella-specific trnD2 gene is shown with its two possible anticodons. Missing data for H. robusta are due to the lack of comparative data. (PDF) [file pone.0155441.s001.pdf]

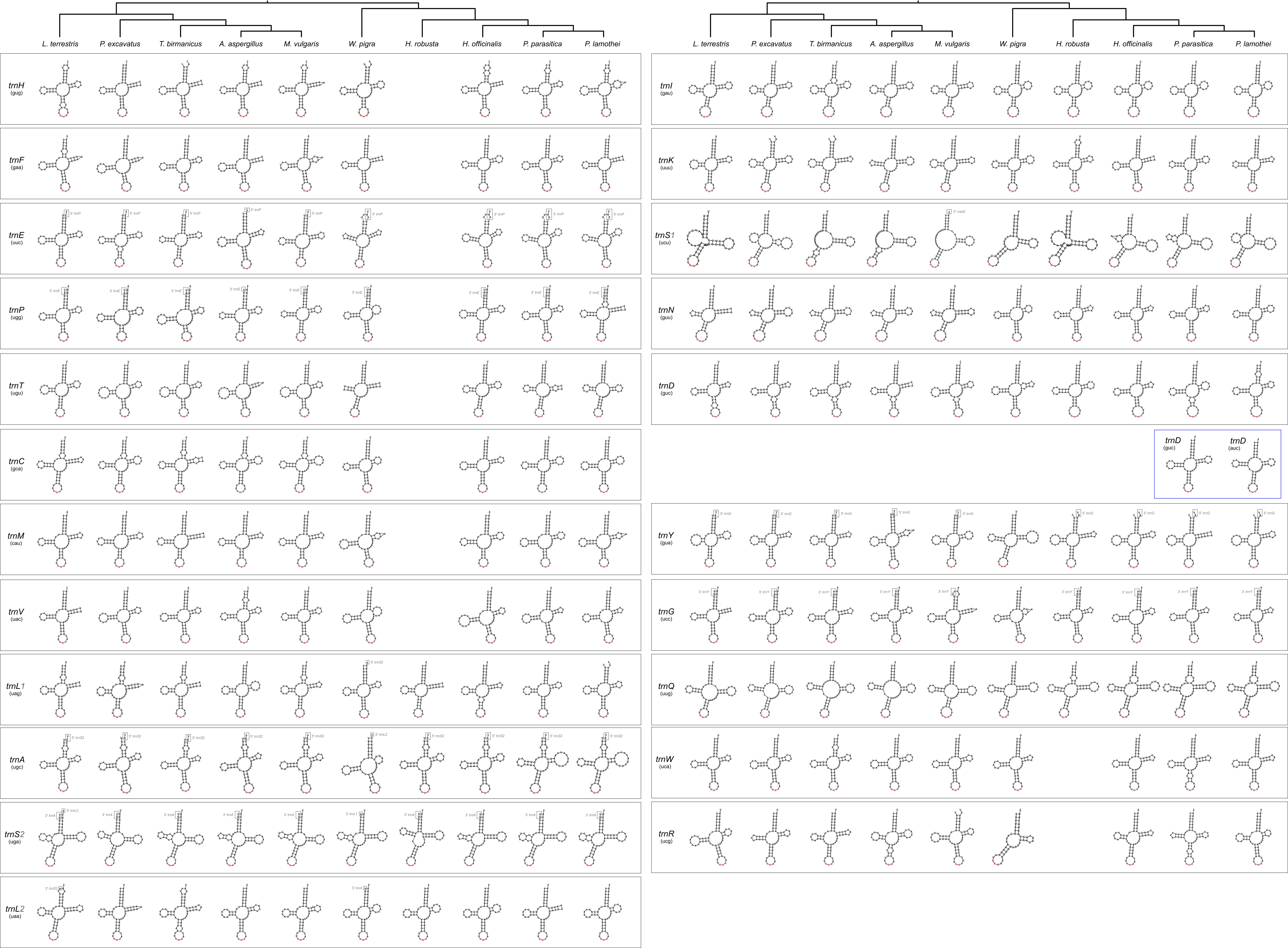

L. terrestris
P. excavatus
T. birmanicus
A. aspergillus
M. vulgaris
W. pigra
H. robusta
H. officinalis
P. parasitica
P. lamothei
trnH (gug)
trnF (gaa)
trnE (uuc)
trnP (ugg)
trnT (ugu)
trnC (gca)
trnM (cau)
trnV (uac)
trnL1 (uag)
trnA (ugc)
trnS2 (uga)
trnL2 (uaa)
trnI (gau)
trnK (uuu)
trnS1 (ucu)
trnN (guu)
trnD (guc)
trnD (guc)
trnD (auc)
trnY (gua)
trnG (ucc)
trnQ (uug)
trnW (uca)
trnR (ucg)
